# Validation of a Kinect V2 based rehabilitation game

**DOI:** 10.1371/journal.pone.0202338

**Published:** 2018-08-24

**Authors:** Mengxuan Ma, Rachel Proffitt, Marjorie Skubic

**Affiliations:** 1 Department of Electrical Engineering and Computer Science, University of Missouri, Columbia, MO, United States of America; 2 Department of Occupational Therapy, University of Missouri, Columbia, MO, United States of America; University of Illinois at Urbana-Champaign, UNITED STATES

## Abstract

Interactive technologies are beneficial to stroke recovery as rehabilitation interventions; however, they lack evidence for use as assessment tools. *Mystic Isle* is a multi-planar full-body rehabilitation game developed using the Microsoft Kinect^®^ V2. It aims to help stroke patients improve their motor function and daily activity performance and to assess the motions of the players. It is important that the assessment results generated from *Mystic Isle* are accurate. The Kinect V2 has been validated for tracking lower limbs and calculating gait-specific parameters. However, few studies have validated the accuracy of the Kinect^®^ V2 skeleton model in upper-body movements. In this paper, we evaluated the spatial accuracy and measurement validity of a Kinect-based game *Mystic Isle* in comparison to a gold-standard optical motion capture system, the Vicon system. Thirty participants completed six trials in sitting and standing. Game data from the Kinect sensor and the Vicon system were recorded simultaneously, then filtered and sample rate synchronized. The spatial accuracy was evaluated using Pearson’s *r* correlation coefficient, signal to noise ratio (SNR) and 3D distance difference. Each arm-joint signal had an average correlation coefficient above 0.9 and a SNR above 5. The hip joints data had less stability and a large variation in SNR. Also, the mean 3D distance difference of joints were less than 10 centimeters. For measurement validity, the accuracy was evaluated using mean and standard error of the difference, percentage error, Pearson’s *r* correlation coefficient and intra-class correlation (ICC). Average errors of maximum hand extent of reach were less than 5% and the average errors of mean and maximum velocities were about 10% and less than 5%, respectively. We have demonstrated that *Mystic Isle* provides accurate measurement and assessment of movement relative to the Vicon system.

## Introduction

In the past decade and quite rapidly in the past five years, Natural User Interfaces (NUIs) and video games have grown in popularity in both consumer applications and in healthcare [1–3]. Specifically, physical rehabilitation (e.g., physical and occupational therapy) has embraced novel NUI applications in clinics, hospitals, nursing homes, and the community [4–6]. Robotic systems have long included game-based and NUI-based user interfaces and most robotic devices provide some form of physical assistance to the patient and/or haptic feedback [7, 8]. With the release of the Nintendo Wii in 2008, many NUI applications for healthcare moved away from bulky, expensive robotics and embraced the portable nature of movement and gesture recognition devices and systems. One of the biggest breakthroughs for this field came in 2010 when Microsoft released the Kinect sensor to accompany its Xbox console system. Within days and weeks of the Kinect’s release, hackers, universities, and companies began to exploit its markerless movement sensing abilities for educational and healthcare use. Since then, there has been an exponential increase in the number of studies that report the use of the Kinect as the input device for a NUI-based rehabilitation game or feedback application [9, 10].

In 2014, Jintronix was the first company to receive FDA approval for its rehabilitation game system that uses the Microsoft Kinect. There are a number of similar companies that utilize the Kinect sensor including SeeMee [11], VirtualRehab [12], Reflexion Health [13], MIRA [14], MotionCare360 [15], and 5Plus Therapy [16]. Many of these systems are marketed for delivering rehabilitation therapy in the home setting. This type of delivery is termed “tele-rehabilitation” and can involve remote monitoring by the therapist or virtual sessions over teleconferencing software [17, 18]. For telerehabilitation or remote sessions, it is imperative that the data the therapist receives from the system or movement-sensing device (such as the Microsoft Kinect) are accurate and reliable. If the therapist plans to use the data for documentation or for reimbursement from a health insurance company, the data ought to be as accurate as current clinical tools (e.g., goniometers).

Only one of the listed companies has validated the measurement capabilities of their systems and of the Microsoft Kinect. Kurillo and colleagues evaluated their system used in 5Plus Therapy against the Impulse motion-capture system (PhaseSpace Inc., San Leandro, CA) and found that it had good accuracy of joint positions and small to large percentage errors in joint angle measurements [19]. However, this study had a small sample size of only 10 subjects and used the first version of the Kinect sensor in its validation. Additionally, the movements used in the assessment were only within a single plane for each movement and all participants were seated during data collection.

Other researchers have validated the Kinect’s measurement and tracking capabilities for both general and specific applications. Hondori and Khademi [20] provide an excellent summary of the work completed prior to 2014. It should be noted that all of these studies evaluated the first version of the Kinect. Following the release of the Kinect V2 sensor, most researchers have focused their validation efforts on gait and posture applications [21–24]. The Kinect V2 has good-to-excellent tracking and measurement capabilities for gait-specific parameters and clinical outcomes. However, many of these studies tracked only the lower limbs. Furthermore, gait is a relatively consistent, rhythmic motion that is consistent across participants, even in rehabilitation populations (i.e., one foot in front of the other). The full-body movements that participants are not limited to specific planes and could choose to use either hand have not been studied in current and prior comparisons of the Microsoft Kinect and optical marker-based motion capture systems.

We have developed software called *Mystic Isle* that utilizes the Microsoft Kinect V2 sensor as the input device [25]. *Mystic Isle* is designed as a rehabilitation game and has shown good results in improving motor function and daily activity performance in persons with chronic stroke [26]. The software initially used the first version (V1) of the Microsoft Kinect as the input device and we completed a study that compared it to the OptiTrack optical system [10]. Based on a visual analysis, we demonstrated that for the hand and elbow, the Kinect V1 has good accuracy in calculating trajectory of movement. For the shoulder, the Kinect V1 tracking abilities limit its validity. Although these findings are promising, the types and number of movements used in the study were limited to those in a seated position and mostly in one plane of movement (e.g., sagittal). Furthermore, the tracking capabilities of the Kinect V2 have substantially improved in the past 7 years and include more data points (joints) for comparison.

The current *Mystic Isle* game involves multi-planar, full body movements. Designed for individuals with diverse abilities, games can be played in a sitting or standing position, depending on the therapy treatment plan. In standing, the player is able to move around in the 3-dimensional space, akin to real-world rehabilitation. Few studies have evaluated the tracking and measurement capabilities of the Microsoft Kinect V2 for full-body, multi-planar movements in both sitting and standing. The purpose of this study was to determine the spatial accuracy and measurement validity of the Microsoft Kinect V2 sensor in a NUI rehabilitation game in comparison to a gold-standard marker-based motion capture system (Vicon^™^).

## Materials and methods

### Participants

Participants were recruited via convenience sample at the University of Missouri- Columbia campus. Participants were included if they: 1) were over the age of 18, 2) could understand conversational English, and 3) had no medical conditions which prevented them from playing video games. The study has been approved by the Health Sciences Institutional Review Board at the University of Missouri with the approval number IRB 2005896 HS. All potential participants were screened and all subjects provided written informed consent before beginning the study.

### Mystic Isle

**Fig 1:**
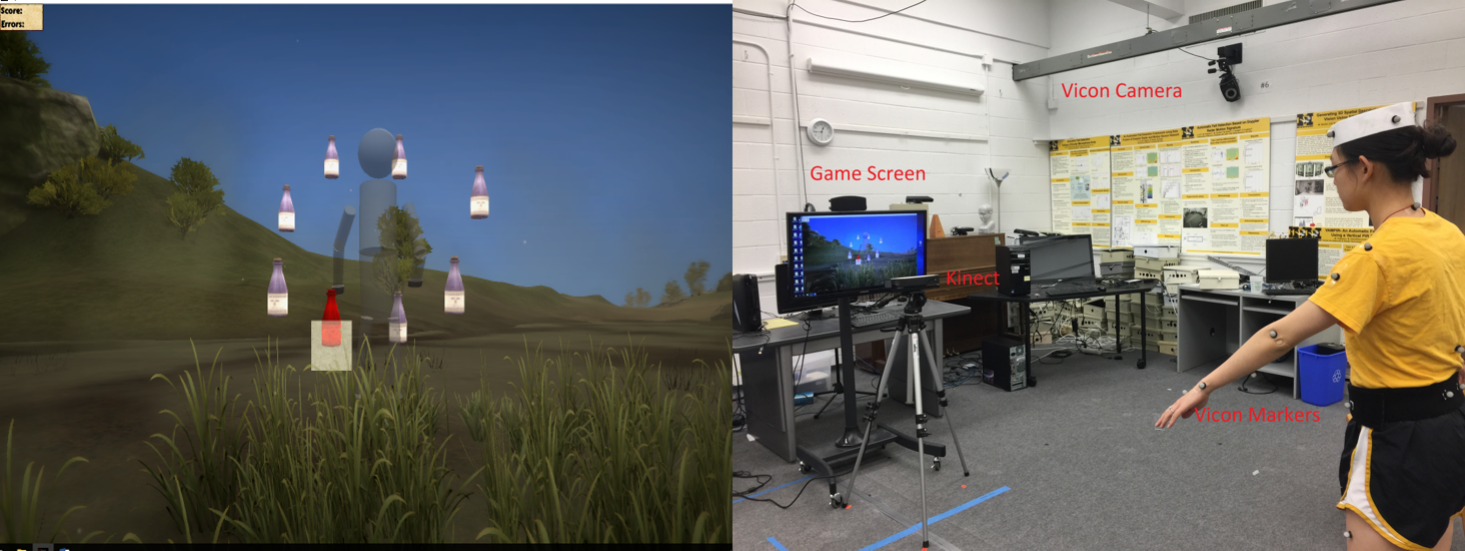
*Mystic Isle* game environment. (a) A virtual avatar collecting targets in a Kinect-based rehabilitation game, *Mystic Isle*. (b) A participant playing the game with Vicon markers on the body. Joint data of game trials were recorded by a Kinect and the Vicon system for validation.

*Mystic Isle* is a platform for rehabilitation that allows a user to interact with a virtual environment by using their body (Fig 1). The *Mystic Isle* software was created in Unity 3D and *Mystic Isle* allows the tracked user to interact with virtual environments and objects in a 3-D world. Using *Mystic Isle*, specific movements, distances, and locations of objects can be tailored to the abilities and requirements of the user. The system uses the Microsoft Kinect V2 camera to track participant movements. The Kinect V2 tracks 20 discrete points/joints on the body of the user. Both gross motor (stepping, jumping, squatting) and fine motor (waving the hand, turning the palm facing up, open/close hand) movements can be tracked. The Kinect V2 tracks the user in 3-dimensional space and then inputs the data in real time to the associated software, *Mystic Isle*. The Kinect V2 tracks and records the x, y, and z coordinates (and confidence) of each discrete joint at either 15 or 30 frames per second.

### Vicon

The Vicon system is a marker-based motion capture system that uses infrared cameras to track the 3-dimensional locations of reflective markers placed on the body. It can be used to measure or give real-time feedback on the movements of the whole body. The Vicon system has been used as an assessment tool for posture analysis, and in balance and reaching studies [27]. It is a gold standard tool for biomechanical kinematic assessment [27]. The sample rate of the Vicon system is 100Hz. For this study, the system included 7 individual cameras placed in a space with a ceiling height of 13 feet.

### Mapping of the joints

**Fig 2:**
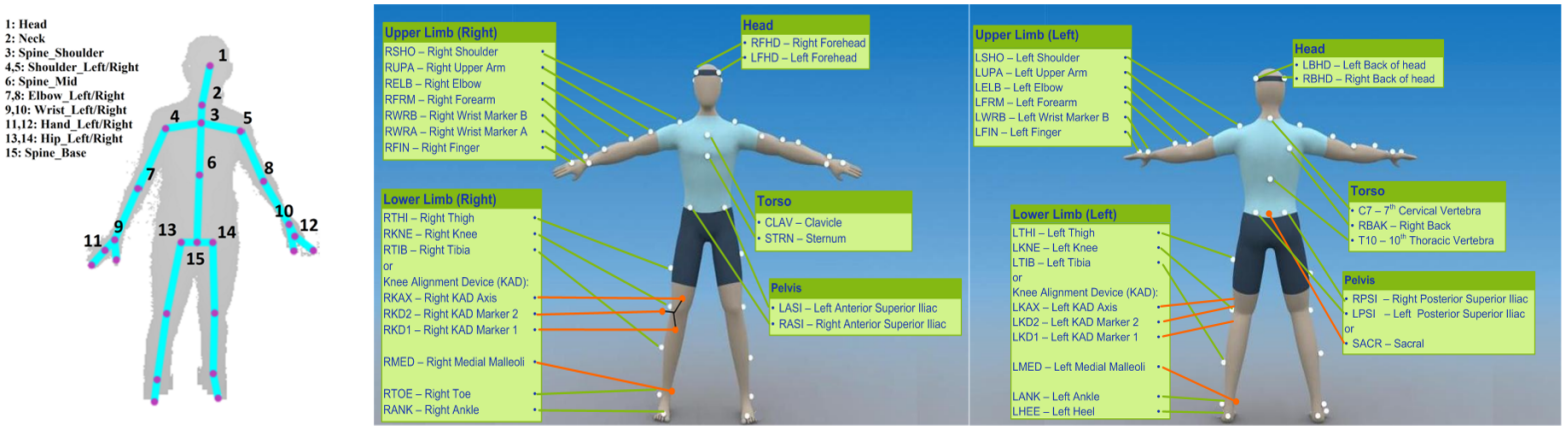
The joint locations of the Kinect V2 skeleton model and a Vicon plug-in gait model. (a) The joint labels and positions of Kinect V2 skeleton model. (b)(c) the marker placement of a Vicon Plug-in gait model. Reprinted from [29] under a CC BY licence, with permission from Vicon Motion Systems.

The Kinect V2 provides a skeleton model [28] (Fig 2(a)) of a game player by recording the x, y, and z coordinates of each discrete joint. The full-body Plug-in Gait model template [29–31] (Fig 2(b) and 2(c)) is commonly used in a Vicon system to build the skeleton model. The joint locations in these two models are not the same. In order to validate the results using joint data from these two skeleton models for this study, we mapped the joints between the two systems (Table 1). For hand, elbow, shoulder and chest joints, the mapping was direct; for the hip and spine base joints, we took the average of several joint locations in the Plug-in gait model to optimize the matching.

**Table 1:** The mapping of joints from the Kinect V2 and the joints from Vicon Plug-in gait model.

Kinect Joints	Vicon Markers	Kinect Joints	Vicon Markers
Hand_Left	LFIN	Hand_Right	RFIN
Elbow_Left	LELB	Elbow_Right	RELB
Shoulder_Left	LSHO	Shoulder_Right	RSHO
Hip_Left^a^	LASI or (LASI+LPSI)	Hip_Right^a^	RASI or (RASI+RPSI)
Spine_Mid	CLAV	Spine_Base^b^	(RASI+LASI) or (RASI+LASI+RPSI+LPSI)

Notes: Multiple mappings to Vicon markers have been tested for these Kinect joints.

^a^The hip joint is mapped to either the ASI marker or the middle position of the ASI and PSI markers.

^b^The Spine_Base joint is mapped to either the middle position of left and right the ASI marker or the middle position of the ASI and PSI markers.

### Data collection

The sampling rate of the Kinect V2 is either 15 or 30 frames per second (f/s), depending on computer performance. In this study, 15 participants’ Kinect V2 data were collected at a rate of 15 f/s on a lower performance laptop computer. The remaining 15 participants’ Kinect V2 data were collected at a rate of 30 f/s on a higher performance desktop computer. In order to investigate how the sample rate influences the accuracy of the measurement outcomes in *Mystic Isle*, we analyzed the errors of extent metrics and speed metrics using the data collected under different frame rates separately. The average difference between the two frame rates in hand extent of reach metrics were 0.70 ± 0.55 centimeters. The average difference between the two frame rates of hand speed metrics were 1.08 ± 1.09 centimeters/second. This variation of errors is tolerable and nearly negligible. Therefore, we will combine samples together for all analyses. The detailed comparison of the accuracies under different sample rates is provided in S1 Fig.

**Fig 3:**
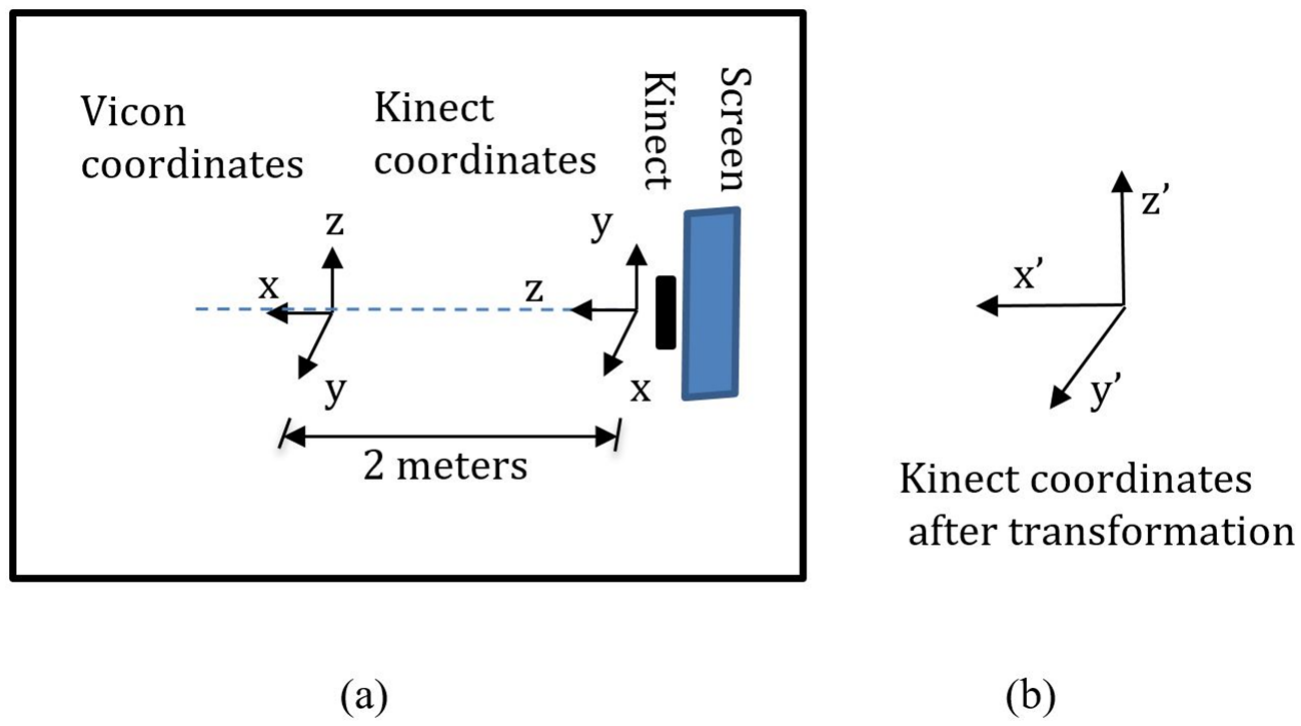
The layout of the data collection room and the coordinate systems of the two systems. (a) The settings of the Vicon system and the Kinect^®^ V2. The origin of the Vicon system is set in the center of the room. The z dimension of the Kinect^®^ V2 coordinate is lined with x dimension of the Vicon’s. (b) The transformed coordinates of the Kinect^®^ V2.

The layout of the data collection room and the coordinate systems of the two systems are displayed in Fig 3. The origin of the Vicon camera system was set to be the center of the room. To make the coordinate space of the Kinect V2 overlay with the Vicon coordinate space, the z dimension of the Kinect^®^ V2 was aligned with the x dimension of the Vicon system (Fig 3). The distance between the origin points of the two systems was 2 meters. The display screen of the game was placed right behind the Kinect V2 and was not occluded by the Kinect V2. Participants stood 1.8-2.4 meters (6-8 feet) from the Kinect V2 and close to the origin point of the Vicon system.

Each participant completed six trials, described below. For each trial, the locations of the virtual objects were determined through a calibration step. We did not instruct participants to use a specific hand for the reaches or foot for stepping (as appropriate).

1. Sitting close: Two rings of eight objects were presented to each participant. The locations of the objects were within arm’s length and no torso movement was required. The subject was seated.
2. Sitting far: Two rings of eight objects were presented to each participant. The locations of the objects required the participant to lean with their torso to be successful. The subject was seated.
3. Standing close: Two rings of eight objects were presented to each participant. The locations of the objects were within arm’s length and no torso movement was required. The subject was standing and did not take a step.
4. Standing far: Two rings of eight objects were presented to each participant. The locations of the objects required the participant to lean with their torso to be successful. The subject was standing and did not take a step.
5. Standing step: Two rings of eight objects were presented to each participant. The locations of the objects required the participant to take a step in order to reach the virtual object.
6. Sorting game: Two rings of eight brightly colored objects were presented to each participant. Four color areas appeared in the virtual environment. The participant was then instructed to select an object and “drag” it into the matching colored area. This game used the same calibration for the “standing close” game.

### Data analysis

Data pre-processing and statistical analysis were performed using R2017a MATLAB. The Kinect V2 coordinates were transformed, data from both systems were filtered and synchronized, and the Vicon data were down sampled. These steps are described in detail below.

#### Coordinate transformation

As shown in Fig 3, the coordinates of the Kinect V2 and the Vicon system are different. In order to visualize the similarities in different dimensions and compute the correlation of the data from the two systems, it was necessary to perform coordinate transformation. We transformed the Kinect V2 coordinates to be the same as the Vicon’s, which means x, y and z dimension of the Kinect V2 Data have been transformed to y, z and x dimension, respectively.

#### Filtering

Noise, such as spike noise, quantization noise and white noise, can be introduced by digital devices when collecting data [32]. In addition, for the Vicon system, marker occlusion is possible and gaps are filled in, introducing noise. To reduce noise, Butterworth filters were applied to the both Kinect V2 and the Vicon data. A sixth-order Butterworth filter with 4Hz cut-off frequency was selected for Vicon data, while a sixth-order Butterworth filter with 3Hz cut-off frequency and was chosen for filtering the Kinect V2 data. The parameters of filters were selected with the largest average Pearson’s r correlation coefficient of the joints, which is also applied in our previous study [33].

#### Synchronization

*Mystic Isle* and the Vicon system started recording data at different times and through different input streams. In order to synchronize the data, the participants clapped three times at the beginning of each trial. The end of the clapping motion was considered to be the start point of a trial and the time stamp of the last game event of *Mystic Isle* was the end of the data trial. The data from two systems were cut based on the start and stop points. Details of synchronizing the data are provided in S2 Fig.

#### Down sampling

The sampling rate of the Vicon system (100 Hz) is different from the sampling rate of the Kinect V2 (15Hz or 30Hz). The velocity metric is affected by different sample rates. Thus, the Vicon data was down sampled close to either 15Hz or 30Hz to match with Kinect V2 data’s. The pseudo code is shown in S1 Table.

### Outcomes

#### Spatiotemporal accuracy

The signals representing the location of joints captured by the Kinect and the Vicon systems are spatial temporal signals. When analyzing the similarity of the spatiotemporal signals from the two systems, the mean of each signal was subtracted from the signal to minimize the bias.

Signal to noise ratio (SNR) compares the level of the ground truth signal with the level of noise. We applied SNR to compare the level of the signals from the Vicon system with the level of the signal difference between the two systems. The formula of SNR is

$$\begin{array}{c} SNR=10{\text{log}}_{10}\frac{Vicon\;data}{Kinect\;data-Vicon\;data}, \end{array}$$ (1)

We averaged the SNR results for each joint in different types of games. SNR is typically computed in decibels (dB). A SNR with 0 dB means the signal and the noise have the same level. A SNR below 0 dB indicates that the noise is larger than the desired signal; a 10 dB SNR indicates that the signal is 10 times larger than the noise [34].

#### Measurement validity

Extent of reach was calculated for each trial. Extent of reach was defined as the distance from the hand joint to the shoulder center, where shoulder center is the middle of the left and right shoulder joints. Suppose the hand joint and the shoulder center are represented by *j*_hand_ = {*h*_x_, *h*_y_, *h*_z_} and *j*_shoulderC_ = {*s*_x_, *s*_y_, *s*_z_}, then the extent of reach for each frame is calculated by

$$\begin{array}{c} Extent\;of\;reach=\sqrt{{\left(h_{x}-s_{x}\right)}^{2}+{\left(h_{y}-s_{y}\right)}^{2}+{\left(h_{z}-s_{z}\right)}^{2}}, \end{array}$$ (2)

We also calculated maximum and mean velocities for each trial. Suppose the hand joint of the *i*^th^ frame is represented by *j*_hand_ = {*x*_i_, *y*_i_, *z*_i_}, the velocity of this frame is calculated by

$$\begin{array}{c} Hand\;Velocity=\frac{\sqrt{{\left(x_{i+1}-x_{i}\right)}^{2}+{\left(y_{i+1}-y_{i}\right)}^{2}+{\left(z_{i+1}-z_{i}\right)}^{2}}}{t_{i+1}-t_{i}}, \end{array}$$ (3)

where *t*, time, is measured and stored automatically with each frame by the Kinect^®^ V2 SDK.

### Statistical analysis

For spatiotemporal accuracy, we calculated the mean Euclidean 3-D distance, Pearson’s *r* correlation coefficient and SNR of each joint to determine the strength of association. For measurement validity, we calculated the difference and percentage error between the two systems for each participant per each game trial. These values were then averaged for different types of games. We also calculated the standard error of the difference, Pearson’s *r* correlation coefficient and intra-class correlation (ICC) with 95 percentage confidence internal.

## Results

### Participants

Thirty subjects participated in this study, including 24 females and 6 males, with an average age of 24.2 years ± 6.6. Only two participants were left handed.

### Spatiotemporal accuracy

#### Upper body

The average correlation coefficient of the arm joints was high; most of the correlation values were above 0.9 (Table 2). In addition, the SNR values of the arm joints (Table 3) were above 5, indicating a signal at least 5 times greater than noise. The hand joints had the greatest correlation between the two systems and very high SNR values. The chest (Spine Mid) “joint” had lower correlation between the two systems along with lower SNR values, ranging from 3 to 10. The mean 3D distance differences of joints were less than 10 centimeters (Table 4). The distance differences of chest (Spine Mid) “joint” were smaller than the joints on the arms. In addition, the distance differences were larger in the “standing step” game where the participants were required to take a step to reach an object.

**Table 2:** Correlation coefficients of spatiotemporal signals from the Vicon and the Kinect V2 for each of the six trials.

Game Types	Sitting Close			Sitting Far			Standing Close			Standing Far			Standing Step			Game		
	r_x_	r_y_	r_z_ ^a^	r_x_	r_y_	r_z_	r_x_	r_y_	r_z_	r_x_	r_y_	r_z_	r_x_	r_y_	r_z_	r_x_	r_y_	r_z_
Upper Body																		
Left Hand	**0.94**	**0.98**	**0.98**	**0.93**	**0.94**	**0.94**	**0.96**	**0.97**	**0.98**	**0.96**	**0.96**	**0.97**	**0.95**	**0.92**	**0.95**	**0.96**	**0.98**	**0.98**
Right Hand	**0.90**	**0.95**	**0.97**	**0.94**	**0.98**	**0.98**	**0.98**	**0.97**	**0.98**	**0.96**	**0.97**	**0.97**	**0.92**	**0.95**	**0.9**4	**0.92**	**0.96**	**0.98**
Left Elbow	**0.94**	**0.97**	**0.97**	**0.96**	**0.96**	**0.96**	**0.96**	**0.96**	**0.96**	**0.96**	**0.95**	**0.95**	**0.93**	**0.91**	**0.92**	**0.97**	**0.97**	**0.94**
Right Elbow	**0.94**	**0.95**	**0.97**	**0.93**	**0.95**	**0.96**	**0.97**	**0.96**	**0.97**	**0.96**	**0.96**	**0.92**	**0.92**	**0.93**	**0.92**	**0.92**	**0.93**	**0.94**
Left Shoulder	0.88	**0.91**	**0.91**	**0.94**	**0.97**	**0.91**	**0.91**	**0.93**	**0.93**	**0.93**	**0.97**	0.84	**0.90**	**0.93**	0.85	**0.96**	**0.98**	**0.93**
Right Shoulder	**0.90**	**0.90**	**0.91**	**0.92**	**0.96**	**0.92**	**0.92**	**0.94**	0.86	**0.92**	**0.97**	0.88	**0.90**	**0.92**	0.87	**0.95**	**0.97**	**0.92**
Spine Mid-CLAV^b^	0.83	0.75	0.85	0.87	0.89	0.84	0.88	0.79	0.78	0.89	0.86	0.79	**0.90**	0.89	0.81	0.68	**0.95**	**0.95**
Lower Body																		
Left Hip-LASI^c^	0.66	0.61	0.60	0.82	0.76	0.57	0.89	0.88	0.55	**0.90**	0.88	0.69	**0.91**	0.89	0.70	**0.90**	0.87	0.87
Right Hip-RASI^c^	0.71	0.61	0.63	0.79	0.77	0.64	0.89	0.83	0.60	0.89	0.87	0.70	**0.92**	0.87	0.64	0.88	0.82	0.71
Left Hip-LASI+LPSI^d^	0.67	0.60	0.63	0.84	0.80	0.59	**0.92**	0.86	0.53	**0.90**	0.89	0.57	0.87	0.89	0.55	**0.90**	**0.92**	0.87
Right Hip-RASI+RPSI^d^	0.69	0.54	0.67	0.77	0.77	0.65	**0.93**	0.73	0.55	**0.91**	0.85	0.69	0.87	0.86	0.63	**0.91**	0.78	0.77
Spine base-RASI+LASI	0.74	0.61	0.62	0.87	0.80	0.43	**0.96**	0.88	0.49	**0.94**	0.88	0.65	**0.91**	0.89	0.66	**0.96**	0.89	**0.91**
Spine base-L/RASI+L/RPSI	0.73	0.58	0.61	0.84	0.81	0.58	**0.95**	0.76	0.63	**0.93**	0.86	0.70	**0.92**	0.87	0.62	**0.95**	0.79	0.79

Notes:

^a^r_x_,r_y_ and r_z_ represent the Pearson’s r correlation coefficient in x, y and z dimensions. Values ≥ 0.90 are bolded. CLAV, L/RASI and L/RPSI are the joint labels from Vicon plug-in-gait model (Table 1).

^b^CLAV represents the clavicle position.

^c^L/RASI represents the left and right anterior superior lilac, and

^d^L/RPSI represents the left and right posterior superior lilac [29].

**Table 3:** Signal-to-noise ratios of spatiotemporal signals from the Vicon and the Kinect^®^ V2 for each of the six trials.

Game Types	Sitting Close			Sitting Far			Standing Close			Standing Far			Standing Step			Game		
SNR	x	y	z^a^	x	y	z	x	y	z	x	y	z	x	y	z	x	y	z
Upper Body																		
Left Hand	**12.4**	**15.2**	**16.5**	**11.6**	**12.8**	**15.0**	**14.7**	**13.3**	17.4	**13.7**	**12.6**	**14.5**	**12.4**	**10.4**	**12.9**	**12.8**	**16.0**	**16.1**
Right Hand	**9.8**	**12.1**	**14.6**	**12.6**	**15.6**	**15.9**	**15.5**	**14.3**	1**9.0**	**13.9**	**13.7**	**15.4**	**10.8**	**11.5**	**11.1**	**11.9**	**14.6**	**15.7**
Left Elbow	**11.3**	**12.7**	**14.6**	**12.7**	**13.2**	**13.2**	**12.9**	**11.0**	**12.5**	**12.2**	**11.1**	**10.5**	**11.1**	**10.3**	**10.0**	**12.1**	**14.4**	**10.0**
Right Elbow	**10.3**	**9.9**	**13.4**	**11.3**	**11.7**	**13.0**	**12.9**	**11.7**	**13.7**	**12.4**	**11.6**	**10.7**	**9.9**	**10.9**	**8.7**	**9.7**	**11.8**	**9.8**
Left Shoulder	6.5	7.5	7.4	**10.3**	**12.1**	7.5	**9.7**	**10.1**	5.3	**10.3**	**12.2**	6.1	**9.4**	**10.8**	6.4	**9.4**	**12.3**	**8.9**
Right Shoulder	7.3	7.4	6.8	**9.6**	**11.1**	**8.2**	**9.2**	**9.5**	5.5	**10.1**	**11.9**	7.6	**8.8**	**10.3**	6.5	**8.7**	**12.8**	**8.2**
Spine Mid-CLAV^b^	4.4	4.1	5.0	7.5	7.9	4.9	**9.0**	5.5	3.6	**9.6**	6.6	5.4	**9.1**	7.9	4.8	3.1	**8.5**	**10.6**
Lower Body																		
Left Hip-LASI^c^	-0.7	-3.1	-3.8	5.5	2.8	-1.0	7.5	**8.2**	-5.4	**8.5**	**8.2**	-3.9	**9.0**	**8.4**	-1.1	7.0	7.9	-2.3
Right Hip-RASI^c^	1.2	-3.5	-7.4	5.1	2.8	-1.2	**8.2**	6.0	-8.9	**8.3**	7.0	-5.4	**9.8**	7.6	-3.7	6.2	5.2	-7.7
Left Hip-LASI+LPSI^d^	-1.3	-5.3	-4.4	5.8	2.4	-0.4	**8.3**	7.9	-5.4	7.7	**8.3**	-5.3	7.3	**8.3**	-1.7	7.1	**8.5**	-3.8
Right Hip-RASI+RPSI^d^	0.2	-5.5	-4.3	4.8	2.0	0.2	**9.0**	4.9	-8.0	**8.1**	6.7	-6.5	7.6	7.2	-4.8	**8.3**	3.9	-9.9
Spine base-RASI+LASI	1.5	-3.3	-8.9	6.9	3.3	-6.2	**12.2**	**8.1**	-7.3	**11.5**	**8.4**	-8.1	**10.1**	**8.5**	-3.4	**10.7**	**8.4**	-3.5
Spine base-L/RASI+L/RPSI	0.8	-4.9	-5.6	6.4	2.5	-1.9	**11.7**	5.4	-8.2	**9.7**	7.0	-8.9	**9.5**	7.5	-5.8	**9.2**	4.3	-9.2

Notes:

^a^x, y and z represent the signal to noise ratio in in x, y and z dimensions. Values > +8 are bolded. CLAV, L/RASI and L/RPSI are the joint labels from Vicon plug-in-gait model (Table 1).

^b^CLAV represents the clavicle position.

^c^L/RASI represents the left and right anterior superior lilac, and

^d^L/RPSI represents the left and right posterior superior lilac [29].

**Table 4:** Spatiotemporal accuracy of joint signals from the Kinect V2 against the Vicon markers for each of the six trials. The accuracy is evaluated by the mean 3D Euclidean distance in centimeter and corresponding standard deviation.

Game Types	Sitting Close	Sitting Far	Standing Close	Standing Far	Standing Step	Game
Diff3D	Mean(SD)	Mean(SD)	Mean(SD)	Mean(SD)	Mean(SD)	Mean(SD)
Upper Body						
Left Hand	4.07(4.11)	4.34(4.05)	3.80(3.69)	5.02(4.52)	8.70(7.21)	6.17(5.34)
Right Hand	4.35(4.36)	5.42(4.81)	3.80(3.37)	5.69(5.53)	9.12(9.57)	8.39(7.02)
Left Elbow	2.80(2.12)	3.07(2.29)	2.92(2.15)	4.60(2.80)	7.19(5.15)	5.69(3.92)
Right Elbow	3.02(2.43)	4.03(2.80)	3.20(2.02)	4.86(3.31)	7.90(6.04)	7.41(5.44)
Left Shoulder	1.87(1.11)	2.39(1.25)	1.97(1.08)	3.47(1.93)	6.19(4.65)	4.71(3.33)
Right Shoulder	1.92(1.09)	2.63(1.43)	2.07(1.12)	3.33(1.90)	6.29(4.62)	4.40(3.18)
Spine Mid-CLAV^a^	1.36(0.65)	2.19(1.03)	1.87(0.85)	2.97(1.51)	6.64(5.09)	5.01(2.76)
Lower Body						
Left Hip-LASI^b^	3.28(1.34)	2.95(0.82)	1.45(0.74)	2.67(1.44)	6.10(4.53)	3.82(2.66)
Right Hip-RASI^b^	1.23(0.53)	1.95(0.77)	1.45(0.74)	2.65(1.46)	6.21(4.38)	4.21(2.94)
Left Hip-LASI+LPSI^c^	2.11(0.91)	2.08(0.73)	1.64(0.82)	2.88(1.51)	5.65(4.09)	4.28(2.62)
Right Hip-RASI+RPSI^c^	1.17(0.53)	1.66(0.73)	1.62(0.80)	2.86(1.43)	5.72(3.87)	4.68(3.05)
Spine base-RASI+LASI	1.93(0.53)	2.05(0.68)	1.21(0.64)	2.38(1.34)	5.29(3.82)	3.35(2.32)
Spine base-L/RASI+L/RPSI	1.44(0.56)	1.61(0.64)	1.45(0.73)	2.66(1.28)	5.29(3.69)	4.24(2.66)

Note: CLAV, L/RASI and L/RPSI are the joint labels from Vicon plug-in-gait model (Table 1).

^a^CLAV represents the clavicle position.

^b^L/RASI represents the left and right anterior superior lilac, and

^c^L/RPSI represents the left and right posterior superior lilac [29].

#### Lower body

When comparing the two systems, the lower body joints (Tables 2 and 3) demonstrated less stability overall showing lower correlation values (0.5 to 0.9) than upper body and large variation in SNR values. However, lower body joints had smaller 3D distance differences than the values of upper body joints (Table 4). The differences were larger when the players performed a step motion in the game trial “standing step”.

### Measurement validity

#### Extent of reach

Overall, the average difference values of maximum extent of reach were less than 3 cm across all six trials and the percentage error was less than five percent (Table 5). More errors were introduced in measurements of the right hand as compared to the left hand. The Pearson’s *r* correlation coefficient of extent of hand in x, y and z dimension are high. Most were greater than 0.8. Only one trial had the lowest value 0.7. Extent of reach in 3D had lower Pearson’s *r* correlation coefficient correlation values compared to extent of reach in each dimension. But the values were not less than 0.6. The intra-class correlation values of extent of reach around the sagittal and frontal axes were very high (>0.96) and larger than movements around the vertical axis for most of the trials. The intra-class correlation values of extent of reach in 3D were relatively low in standing-type trials.

**Table 5:** Accuracy of clinical measures from the Kinect V2 against the Vicon for six types of game trials. Accuracies were validated using mean difference, standard error, mean percentage error, Pearson’s *r* correlation coefficient and intra-class correlation with corresponding 95 percentage confidence internal.

	Difference		SE^b^		Percentage error		Peason’s r		ICC(3,1)	
Metrics	Left	Right^a^	Left	Right	Left	Right	Left	Right	Left	Right
Sitting close										
Max Ext_x	0.8	2.4	0.2	0.3	1.9	5.4	1.0	1.0	0.99(0.99;1.00)	0.97(0.95;0.99)
Max Ext_y	1.8	1.9	0.4	0.5	3.2	3.2	1.0	1.0	0.98(0.91;0.99)	0.98(0.97;0.99)
Max Ext_z	1.4	3.0	0.4	0.4	2.6	5.4	0.9	0.8	0.93(0.84;0.96)	0.86(0.71;0.93)
Max Ext_3D	1.7	2.3	0.3	0.3	2.4	3.3	0.9	0.8	0.85(0.92;0.95)	0.85(0.37;0.95)
Max Speed	6.7	5.0	1.4	0.9	3.1	2.5	1.0	1.0	0.99(0.97;1.00)	1.00(0.99;1.00)
Mean Speed	2.0	2.0	0.3	0.3	12.0	10.6	1.0	1.0	0.99(0.81;1.00)	0.98(0.75;1.00)
Sitting far										
Max Ext_x	2.1	2.9	0.5	0.4	4.5	6.1	1.0	1.0	0.98(0.93;1.00)	0.97(0.83;0.99)
Max Ext_y	1.3	2.4	0.3	0.3	2.4	4.0	1.0	1.0	1.00(0.99;1.00)	0.98(0.95;0.99)
Max Ext_z	1.0	2.8	0.4	0.4	1.9	5.0	1.0	0.9	0.97(0.94;0.99)	0.93(0.85;0.96)
Max Ext_3D	1.8	2.7	0.3	0.4	2.6	3.9	1.0	0.8	0.97(0.89;0.99)	0.81(0.42;0.92)
Max Speed	3.3	4.7	0.5	1.0	2.4	2.8	1.0	1.0	1.00(1.00;1.00)	1.00(0.99;1.00)
Mean Speed	2.4	2.0	0.3	0.3	12.9	11.0	1.0	1.0	1.00(0.99;1.00)	0.98(0.94;0.99)
Standing close										
Max Ext_x	1.8	2.7	0.3	0.3	3.5	5.3	1.0	1.0	0.99(0.98;1.00)	0.98(0.96;0.99)
Max Ext_y	1.2	2.3	0.4	0.3	1.9	3.7	1.0	0.9	0.99(0.98;1.00)	0.96(0.91;0.98)
Max Ext_z	1.0	2.7	0.4	0.4	1.6	4.2	0.8	0.8	0.88(0.74;0.95)	0.83(0.37;0.94)
Max Ext_3D	1.6	2.5	0.3	0.5	2.3	3.5	0.9	0.6	0.90(0.68;0.96)	0.74(0.45;0.88)
Max Speed	7.6	7.4	1.2	1.4	4.1	4.3	1.0	1.0	0.99(0.99;1.00)	0.99(0.98;1.00)
Mean Speed	1.4	1.7	0.3	0.3	6.5	7.5	1.0	1.0	0.99(0.93;1.00)	0.99(0.94;0.99)
Standing far										
Max Ext_x	2.7	2.9	0.5	0.3	4.8	4.9	1.0	0.9	0.99(0.96;1.00)	0.93(0.74;0.97)
Max Ext_y	1.0	1.4	0.2	0.2	1.7	2.4	1.0	1.0	0.99(0.99;1.00)	0.99(0.98;1.00)
Max Ext_z	1.1	2.9	0.4	0.4	1.7	4.6	0.9	0.9	0.94(0.85;0.97)	0.92(0.69;0.97)
Max Ext_3D	2.5	2.6	0.5	0.4	3.5	3.6	0.7	0.6	0.74(0.32;0.89)	0.68(0.18;0.86)
Max Speed	5.9	8.5	1.0	1.2	3.3	3.9	1.0	1.0	1.00(0.99;1.00)	0.99(0.97;0.99)
Mean Speed	2.1	2.0	0.3	0.3	9.5	9.0	1.0	0.9	0.97(0.93;0.99)	0.97(0.94;0.99)
Standing step										
Max Ext_x	2.6	3.0	0.4	0.3	4.9	5.4	1.0	1.0	0.99(0.97;1.00)	0.97(0.89;0.99)
Max Ext_y	0.9	2.3	0.2	0.4	1.6	3.5	1.0	0.9	0.99(0.99;1.00)	0.97(0.93;0.98)
Max Ext_z	1.8	2.8	0.4	0.4	2.8	4.5	0.9	0.9	0.88(0.62;0.95)	0.90(0.41;0.97)
Max Ext_3D	2.3	2.5	0.3	0.4	3.4	3.5	0.8	0.7	0.87(0.69;0.94)	0.75(0.46;0.88)
Max Speed	6.6	4.9	1.1	1.0	3.8	2.3	1.0	1.0	1.00(1.00;1.00)	1.00(0.99;1.00)
Mean Speed	1.6	1.7	0.3	0.3	4.9	5.5	1.0	1.0	1.00(0.99;1.00)	0.99(0.96;1.00)
Game										
Max Ext_x	1.5	3.0	0.3	0.3	2.8	5.0	1.0	0.9	0.99(0.98;1.00)	0.91(0.59;0.97)
Max Ext_y	1.8	2.8	0.4	0.4	3.2	4.7	1.0	0.9	0.98(0.96;1.00)	0.96(0.91;0.98)
Max Ext_z	2.8	2.7	0.4	0.5	4.2	4.4	0.7	0.8	0.70(-0.10;0.89)	0.87(0.73;0.94)
Max Ext_3D	2.4	2.9	0.5	0.5	3.4	4.1	0.8	0.6	0.81(0.51;0.92)	0.63(0.18;0.83)
Max Speed	8.1	9.3	1.6	1.7	4.7	4.5	1.0	1.0	0.99(0.98;1.00)	0.99(0.97;0.99)
Mean Speed	3.5	3.7	0.6	0.7	9.2	9.0	0.9	0.9	0.97(0.93;0.98)	0.97(0.94;0.97)

^a^‘Left’ and ‘Right’ means left hand and right hand.

^b^‘SE’ means standard errors.

#### Maximum and mean velocity

Maximum velocity had larger errors than mean velocity over all the trials (Table 5). The largest average error of maximum velocity was about 10 cm/s from the “game” trial. For mean velocity, the largest amount of error was less than 4 cm/s. When considering percentage error, the average percentage error of mean velocity was about 10% and the average percentage error of maximum velocity was less than 5%. The errors from the “game” trial were greater than other trials and mean velocity errors were larger in sitting versus standing trials. The Pearson’s *r* correlation coefficient values of maximum and mean velocities were not less than 0.9 and the intra-class correlation values were not less than 0.97.

## Discussion

*Mystic Isle*, similar to other rehabilitation-focused games and software, has been shown feasible as an intervention for people with stroke with the Microsoft Kinect V2 camera being used as an input device. Before using the Kinect V2 and the *Mystic Isle* software as an assessment tool in a clinical setting, it is necessary to validate the accuracy of the Kinect V2’s tracking capabilities. Therefore, the purpose of this study was to determine the spatial accuracy and measurement validity of the Microsoft Kinect V2 sensor in a rehabilitation game in comparison to a gold-standard marker-based motion capture system (Vicon). We have demonstrated that *Mystic Isle* provides an accurate measurement of movement relative to the Vicon system; however there are some movements and planes of measurement in which the accuracy is considerably lower. The findings from this study are similar to findings from other comparison studies between the Kinect V1 and the Vicon. This study is different from prior work in that we tracked movements of the upper limbs during unrestrained full-body movements (versus just the lower limbs during walking) and the participants were not limited to specific planes of movements and could choose to use either hand during a reach [32, 34, 35]. The movements in this study more closely mimic real-world performance; this has significant implications for clinical rehabilitation practice. Each of these points is discussed below. We conclude with limitations and next steps for research and clinical practice.

With regards to exploring measurement validity, we found that the errors of hand extension and speed metrics from the right hand were larger than the errors of the left hand, but the higher error rate is still close enough for relevant clinical assessments. Also, we observed that the percentage errors of mean velocity of sitting trials were larger than the error from standing trials. In sitting, participants tended to move slower than in standing; thus, overall velocity was lower in sitting. However, the absolute errors were similar across all trials. The sixth trial, the sorting game, had the largest percentage errors of all trials. There are two reasons for this. First, the sorting game trial was the longest trial. The longer a person is engaged with the task, the greater the potential for noise to be introduced. Second, the required movements for game success were different than the other trials. Participants “dragged” a virtual target from one side of the screen to another in order to “sort” the virtual objects. Further, some participants had to bend at the knee in order to “place” the virtual object in the correct spot. The bending position likely introduced some noise and limited the tracking capability of the Kinect V2, particularly at the hip joints.

When considering spatial accuracy of the tracked joints, the joints of the arm were highly correlated between both systems and had high SNR values. The joints of the hip had much lower SNR values and fewer correlations over 0.90. Other researchers have reported similar findings with regards to the lower body [34]. Mentiplay et al. found poor agreement between Kinect V2 and a Vicon system in peak hip flexion [36]. These lower correlation values have often been interpreted as a consequence of the optimization of Kinect SDK for gesture-based games [34]. Thus, the Kinect SDK appears to provide higher tracking abilities on upper body joints.

Despite the decreased spatiotemporal accuracy of the Kinect for tracking lower body joints, researchers have shown that the Kinect is able to track walking paths and provide data for calculating gait-related variables with relatively high accuracy (e.g., stride length, walking speed) [24, 37]. Guess et al. showed the Kinect can accurately measure hip and knee flexion angles for a vertical drop jump [35]. One of the first evaluations of the Kinect for upper body tracking demonstrated similar percentage errors [32]. In this study, we explored full-body movements that involved reaching, sitting, stepping, and cross-body movements. These results add a richness to the primarily gait-related literature validating the use of the Kinect for tracking upper body kinematics during full body movement. Allowing participants more freedom in a reaching movement (e.g., choice of hand, allowing cross-body reaches) mimics daily activity much more closely than other studies [32, 34, 35]. This may limit the internal validity of the study; however, it greatly increases the external validity of the findings. We are the first to validate the Kinect V2 in this scenario.

Additionally, these findings support the use of the Kinect V2 in a clinical rehabilitation setting. We have shown that the Kinect V2 is an accurate tool for tracking movement; the clinical measurements we can obtain (e.g., extent of reach) are repeatable and valid. Reliability of standard clinical assessment tools for range of motion (goniometers) vary across clinical populations and joints measured [38, 39]. The ICCs between raters and between tools in prior studies range from 0.50 to 0.98 [40, 41]. Therefore, the Kinect V2 has the potential to be utilized in clinical practice and home-based rehabilitation to complement existing outcome assessments. Furthermore, these data can be collected by the Kinect V2 in remote settings, such as a patient’s home, and provide clinicians with a look at performance over time. Health insurance companies are demanding more data and metrics to support clinical decision making. With a validated sensor, this system has the potential to provide rehabilitation clinicians and insurers with high quality, performance-based data and outcomes.

This study has a few limitations. First, the sample is relatively homogenous, young, and a majority of females, which limits a generalization of the findings to an older population, which is common in stroke rehabilitation. Our previous studies with the *Mystic Isle* game have involved stroke patients [25, 42]; however, we have not yet validated the assessments in this population. Our ongoing research is investigating this further in an older population. Second, there were some error differences in tracking the left and right hands, although these were not statistically significant and the errors are within acceptable rates for clinical use for both left and right sides [37, 43]. Lastly, people with stroke have different movement patterns and postures as compared to healthy individuals. Flexor synergy patterns and spasticity might make it more difficult for the Kinect V2 to reliably track the more affected extremity; however, we have had much success in our prior work [25, 42].

Our preliminary research has shown that motor function and daily activity performance of stroke patients can improve through the use of *Mystic Isle* as an in-home intervention [26]. The next step for our research is to use the Kinect V2 to complete assessments of movements in people with stroke. Additionally, we are building an in-home monitoring system that utilizes the Kinect V2 for ambient tracking of movement and as an assessment of upper extremity movement performance. These studies will further test the use of the Kinect V2 as a valid tool for tracking movements in rehabilitation populations.

## Supporting information

S1 Fig Comparison of measurement validity under difference sample rates. The influence of the sample rates of the Kinect to the accuracy of clinical measurement were investigated.
(PDF)

S2 Fig Start and stop points for synchronization. The figure illustrates the starting point and stop point on one subject’s data.
(PDF)

S1 Table Pseudocode for preprocessing the joint data from the Kinect and Vicon system. The data was preprocessed by R2017a MATLAB. The Kinect data were transformed, filtered and synchronized. The Vicon data were synchronized and down-sampled.
(PDF)

S1 Dataset Spatiotemporal accuracy results. (ZIP)

S2 Dataset Validation results of extent of reach metrics. (XLSX)

S3 Dataset Validation results of max and mean speed metrics. (XLSX)
