# Supplementary material for: Validation of a Kinect V2 based rehabilitation game
Source: PLoS One. 2018 Aug 24;13(8):e0202338. doi: 10.1371/journal.pone.0202338 (PMC6108894; doi:10.1371/journal.pone.0202338)
Supplement: S1 Fig — The influence of the sample rates of the Kinect to the accuracy of clinical measurement were investigated. (PDF) [file pone.0202338.s001.pdf]

Comparison of measurement validity under difference sample rates

The sample rate of a Kinect V2 sensor is either 15 or 30 frames per second(f/s). We conducted a study to investigate the influence of the sample rates to the accuracy of clinical measurements. In the study, we collected data from 15 participants at a rate of 15 f/s of the Kinect and 100 f/s of the Vicon system and collected data from another 15 participants at a rate of 30 f/s of the Kinect and 100 f/s of the Vicon. Then, we evaluated the accuracy of extent of reach and speed metrics of each participant for each game trial. Next, we averaged the errors under the same sample rate for different games. At last, we compared the difference of the average errors in different sample rates. The differences of average errors of extension and speed metrics under two sample rates are shown in figure 1 and 2, respectively. The average difference between the two frame rates in hand extent of reach metrics was 0.70 ± 0.55 centimeters. The average difference between the two frame rates of hand speed metrics was 1.08 ± 1.09 centimeters/second. This variation of errors is tolerable and nearly negligible.

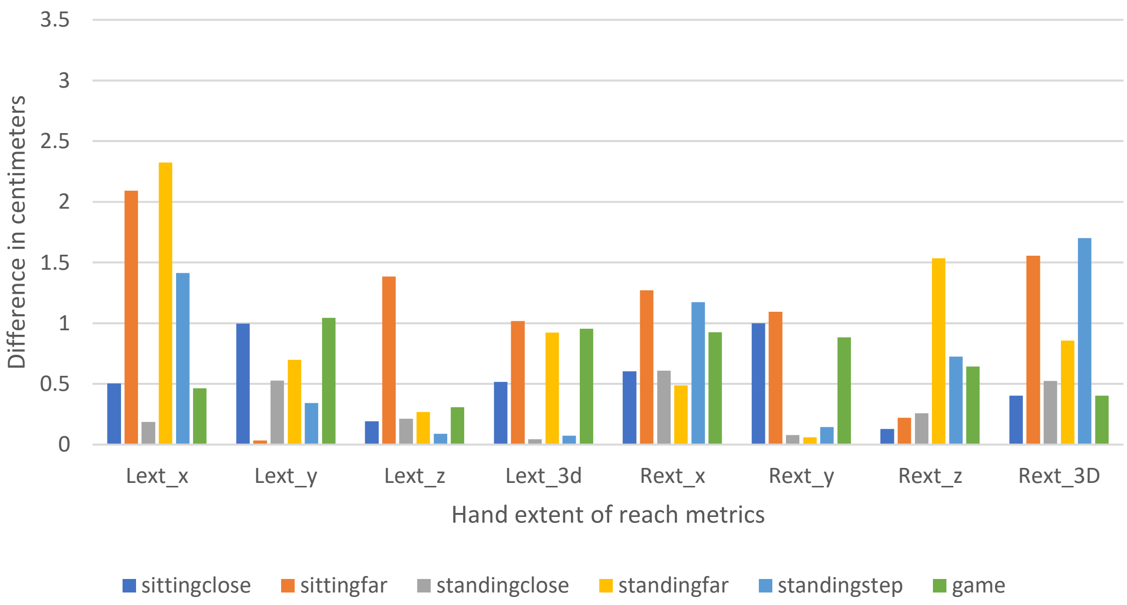

Figure 1 The absolute differences of average errors of extension metrics under two sample rates for different game trials. The overall difference for all the participants and all the games was 0.70±0.55 cm.

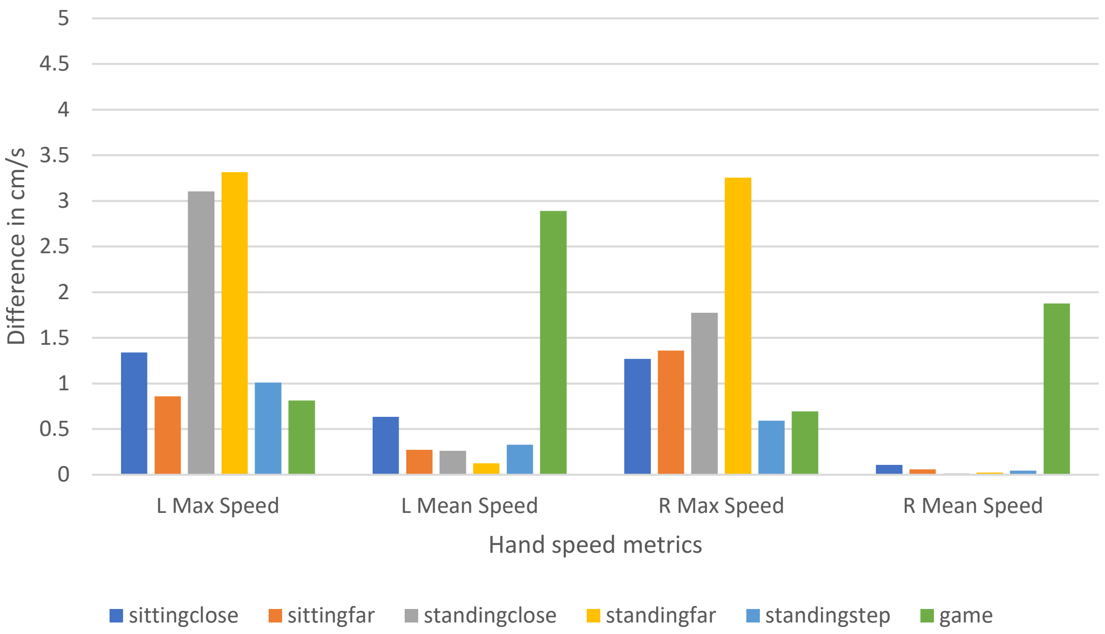

Figure 2 The absolute differences of average errors of speed metrics under two sample rates. The overall difference for all the participants and all the games was 1.08±1.09 cm/s.
