# Supplementary material for: Validation of a Kinect V2 based rehabilitation game
Source: PLoS One. 2018 Aug 24;13(8):e0202338. doi: 10.1371/journal.pone.0202338 (PMC6108894; doi:10.1371/journal.pone.0202338)
Supplement: S2 Fig — The figure illustrates the starting point and stop point on one subject’s data. (PDF) [file pone.0202338.s002.pdf]

Start and Stop points for Synchronization

The Kinect and Vicon system may start recording the game data at the different time. Players were performed three claps before playing each game for synchronization purpose. The start point of a game trial is the end of the clapping motion. In the hand trajectories of each dimension, the three peaks at the beginning represent the clapping motion. We considered the third peak (marked as ‘*’ in Figure S1) of the left-hand trajectory in Y dimension as the end of the clapping motion, which is also the start point of the game. Mystic Isle generates the event log. We considered the timestamp of the last event as the end of the game trial which is marked as ‘o’ in Figure S1.

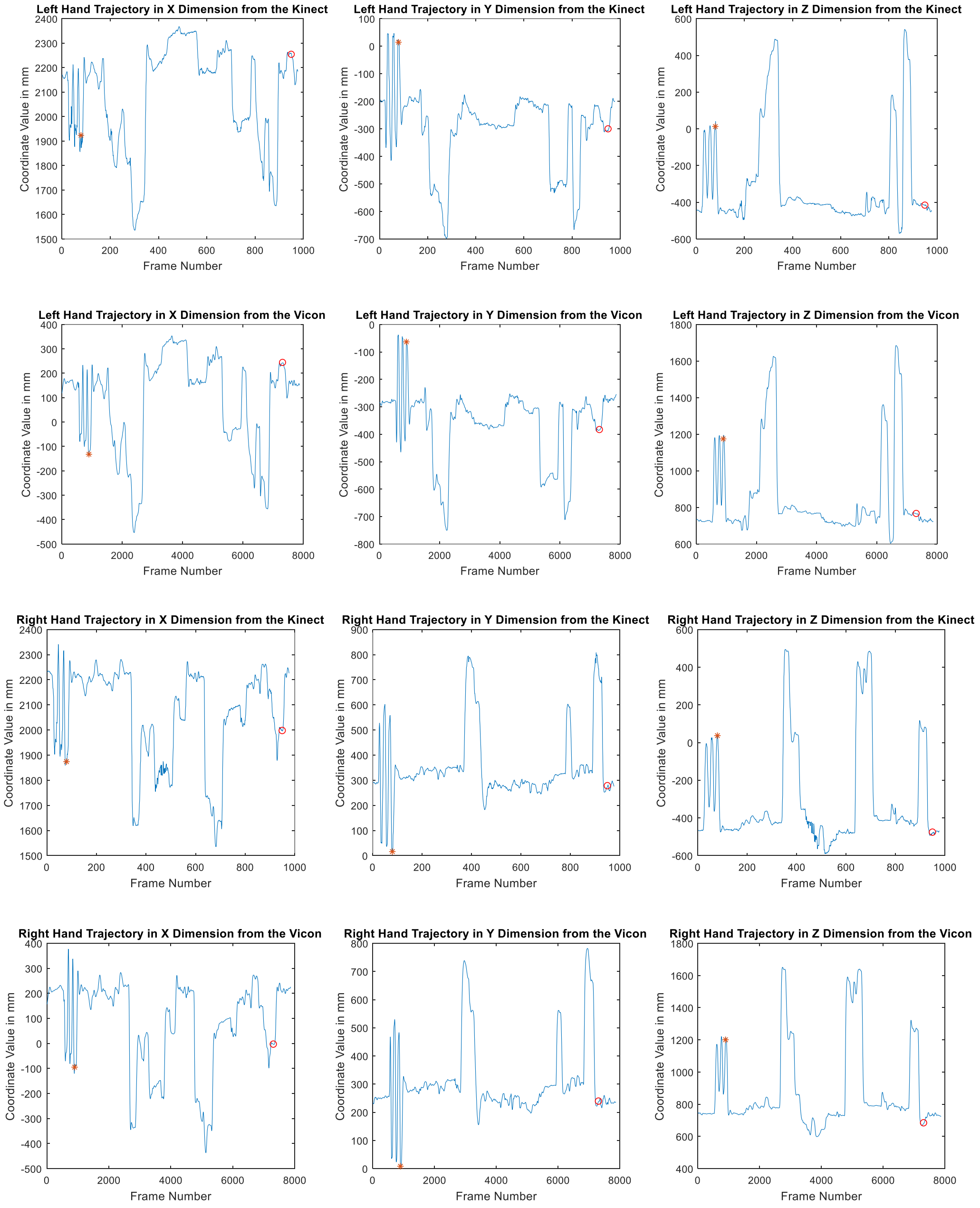

Figure S1 Left and Right-Hand Trajectories of Subject 8 standing far game. The third peak (marked as ‘’) of the left-hand trajectory in Y dimension is the start point of the game. The timestamp of the last event (marked as ‘o’) is the end of the game trial.*
