# Supplementary material for: Validation of a Kinect V2 based rehabilitation game
Source: PLoS One. 2018 Aug 24;13(8):e0202338. doi: 10.1371/journal.pone.0202338 (PMC6108894; doi:10.1371/journal.pone.0202338)
Supplement: S1 Table — The data was preprocessed by R2017a MATLAB. The Kinect data were transformed, filtered and synchronized. The Vicon data were synchronized and down-sampled. (PDF) [file pone.0202338.s003.pdf]

S1 Table Pseudocode for preprocessing the joint data from the Kinect and Vicon system. The data was preprocessed by R2017a MATLAB. The Kinect data were transformed, filtered and synchronized (Line 1-17). The Vicon data were synchronized and down-sampled (Line 19-33). Butter, filter, floor and downsample are MATLAB functions.

```
1:   Begin the Kinect data pre-processing procedure
2:        Load the Kinect data to array k_x, k_y and k_z
3:          % Data transformation for Kinect joint data
4:        Tran_x ← k_z
5:        Tran_y ← k_x
6:        Tran_z ← k_y
7:        For the joint data in each dimension
8:              % Filter the data
9:            Order = 6; f_cut = 3; f_sample = 15 or 30
10:           [B,A] = butter(order,2* f_cut / f_sample);
11:           flt_data = filter(B,A,data);
12:             % Synchronization
13:           Start_point = end of the clipping motion
14:           Stop_point: timestamp of the last game event
15:           cut_data = flt_data(start:stop);
16        end For Loop
17:  end procedure
18:
19:  Begin the Vicon data pre-processing procedure
20:      Load the filtered Vicon data to array v_x, v_y and v_z
21:      % Vicon data has been filtered using Vicon software Nexus
22:      For the joint data in each dimension
23:             % Synchronization
24:           Start_point = end of the clipping motion
25:           Stop_point: timestamp of the last game event
26:           cut_data = data(start:stop);
27:             % Down sample
28:           Kinect_samples = number of samples of cut_data from Kinect
29:           Vicon_samples = number of samples of cut_data from Vicon
30:           DwSampleRate = floor (Vicon_samples / Kinect_samples)
31:           DwSample_data = downsample (cut_data, DwSampleRate);
32        end For Loop
33:  end procedure
```
